# Information stored in memory affects abductive reasoning

**DOI:** 10.1007/s00426-020-01460-8

**Published:** 2021-01-11

**Authors:** Anja Klichowicz, Daniela Eileen Lippoldt, Agnes Rosner, Josef F. Krems

**Affiliations:** 1 Department of Psychology, Chemnitz University of Technology, Chemnitz, Germany; 2 Department of Psychology, University of Zurich, Zurich, Switzerland

## Abstract

Abductive reasoning describes the process of deriving an explanation from given observations. The theory of abductive reasoning (TAR; Johnson and Krems, Cognitive Science 25:903–939, 2001) assumes that when information is presented sequentially, new information is integrated into a mental representation, a situation model, the central data structure on which all reasoning processes are based. Because working memory capacity is limited, the question arises how reasoning might change with the amount of information that has to be processed in memory. Thus, we conducted an experiment (*N* = 34) in which we manipulated whether previous observation information and previously found explanations had to be retrieved from memory or were still visually present. Our results provide evidence that people experience differences in task difficulty when more information has to be retrieved from memory. This is also evident in changes in the mental representation as reflected by eye tracking measures. However, no differences are found between groups in the reasoning outcome. These findings suggest that individuals construct their situation model from both information in memory as well as external memory stores. The complexity of the model depends on the task: when memory demands are high, only relevant information is included. With this compensation strategy, people are able to achieve similar reasoning outcomes even when faced with tasks that are more difficult. This implies that people are able to adapt their strategy to the task in order to keep their reasoning successful.

## Introduction

Inferring an explanation from a set of observations is one of the most challenging tasks our minds engage in every day. This process is called abductive reasoning (Johnson & Krems, 2001; Peirce, 1931) and is understood as one out of three classes of inference (abduction, deduction, induction, see Table 1, for an overview see Peirce, 1931). In deduction a rule (If E → O) and the explanation (E) is present, and the data (or observation; O) have to be inferred. In induction the explanation as well as data (observations) is present and one has to infer the rule. In contrast, in abduction an explanation is derived from observations given a rule (Josephson & Josephson, 1996; Meder & Mayrhofer, 2017; Peng & Reggia, 1990).

**Table 1:**
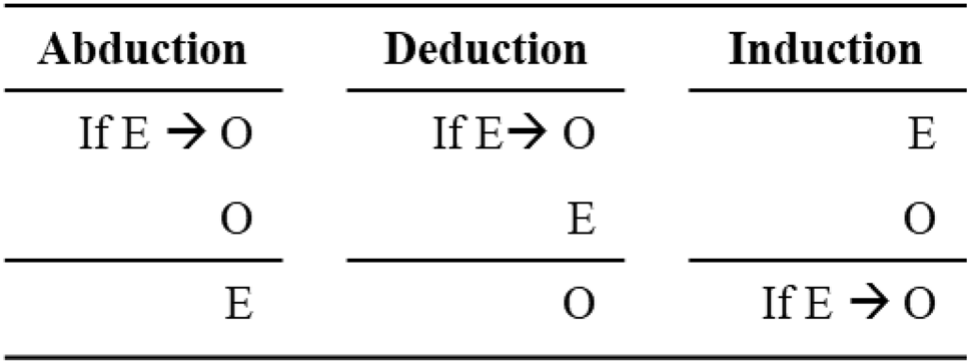
Overview over abduction, deduction, induction based on a rule (If E → O), an observation (O), and an explanation (E)

It has been shown that finding the explanation can further understanding (Lombrozo, 2006), it facilitates learning (Murphy & Allopenna, 1994; Williams & Lombrozo, 2010), can influence our judgments in terms of the perceived typicality of category members (Ahn, Marsh, Luhmann, & Lee, 2002; Murphy & Allopenna, 1994), and foster conceptual coherence (Murphy & Medin, 1985; Patalano, Chin-Parker, & Ross, 2006). Having explanations available puts us in a better position to predict and control the future (Lombrozo, 2006). Therefore, understanding how people infer that one explanation is more likely than another is central in understanding human thinking.

Abduction is a highly complex process because observations can lead to a combination of different explanations, but a single explanation can also account for a number of observations (Johnson & Krems, 2001). The best explanation is defined as the explanation with the lowest level of complexity. For example, when physicians attempt to infer a diagnosis for a number of symptoms, they have to integrate all the available information, some of which is not currently visible but may have to be recalled from patient reports, examinations, or laboratory tests.

Research on eye movements has found that, when retrieving information that was previously encoded at a specific spatial position, a person’s gaze, and respectively, the focus of attention, returns to this spatial location as an aid to working memory (Johansson & Johansson, 2014, 2020; Scholz, Klichowicz, & Krems, 2018). To remember all the relevant information, a physician might, therefore, look at the patient’s file without even opening it, using eye movements to facilitate memory retrieval. This example shows that not only online reasoning skills but also memory plays an important role in abductive reasoning, and that eye movements are used to access memory contents, even if no information is visible in the visual array (for overviews see Ferreira, Apel, & Henderson, 2008; Richardson, Altmann, Spivey, & Hoover, 2009; Wynn, Shen, & Ryan, 2019). It also shows the complexity of the task, as a high number of symptoms can result in different combinations of diagnoses.

A number of process models have been developed to describe the process of abductive reasoning (e.g., TAR: Johnson & Krems, 2001; HyGene: Thomas, Dougherty, Sprenger, & Harbison, 2008). In this study, we focus on the Theory of Abductive Reasoning (Johnson & Krems, 2001), which describes the process of abductive reasoning comprehensively and not just parts thereof.

### The reasoning process

TAR (Johnson & Krems, 2001) assumes that during sequential information presentation, new observations are integrated into a mental representation called a situation model. New information is thus assigned to slots in memory which, taken together, form an overall understanding of the current situation. Research has found evidence that this situation model is located in working memory (Böhm & Mehlhorn, 2008; Thomas, Dougherty, Sprenger, & Harbison, 2008). TAR (Johnson & Krems, 2001) calls this step *comprehend*. In a previous study, we found that this situation model expands over the sequential presentation of observations, becoming more complex over the course of a reasoning task (Klichowicz, Strehlau, Baumann, Krems, & Rosner, 2020). That is, as more information has to be integrated, the number of spatial areas that are associated to explanations grows. As people gaze back at locations that contained information that has to be retrieved, the number of spatial areas looked at grows as well.

To form a correct situation model, is it necessary to have all previous information active in memory, according to research showing that the generation of explanations is influenced by the knowledge currently activated in memory (Mehlhorn, Taatgen, Lebiere, & Krems, 2011; Rebitschek, Krems, & Jahn, 2016; Thomas, Dougherty, Sprenger, & Harbison, 2008). Based on the situation model, the reasoner forms explanations of the observation. If able to form concrete explanations, the reasoner executes a *consistency check* in terms of the implications of these explanations for the situation model. If a combination of explanations can explain all observations without any discrepancy or redundancy, the process is successful. Tracking participants’ eye movements during abductive reasoning indeed showed that observations as well as explanations must be part of the situation model as participants look at both locations during the retrieval of these information to make an inference (Klichowicz, Strehlau, Baumann, Krems, & Rosner, 2020).

However, our results revealed that explanations receive much more attention than observations. This can be explained by the fact that explanations may be subject to change throughout the reasoning process, whereas an observation does not change once it has been made. This also shows that explanations that have already been found have more weight in the overall explanation of a set of observations. Following this argument, we assume that participants put more effort into keeping information that is highly relevant for the final explanation active. Observations might lose their relevance as soon as they are concretely explained, that is, their activation might decline.

Other research by Bauman et al. has shown that there are different levels of activation in working memory during abductive reasoning (Baumann, Mehlhorn, & Bocklisch, 2007). They found that explanations that are relevant for explaining current observations are kept in a more active state than irrelevant explanations. That is, information in the situation model is more active the more relevant it is to the process of reasoning. This is also due to limited working memory capacity (Baddeley & Hitch, 1974; Johnson-Laird, Byrne, & Schaeken, 1992).

To integrate new information into the situation model, one has to retrieve information that is already contained in the model. As the retrieval of information absorbs resources (Hayhoe, Bensinger, & Ballard, 1998), people only engage in active memorization and retrieval when necessary. This is also illustrated by a study by Ballard, Hayhoe, and Pelz (1995), who asked their participants to copy a pattern of colored blocks. They found that participants kept the requirements for memory as low as possible by using more eye movements to gather information from the environment when needed. In their study, participants never used memorization and retrieval as a strategy.

The aforementioned research suggests that a task is perceived as more difficult when the demands on working memory are high. This should also have an impact on the outcome of the reasoning process, as only information that is represented can be taken into account in seeking an explanation. As stated earlier, the situation model as proposed by TAR (Johnson & Krems, 2001) is highly dependent on information that is active in memory (Mehlhorn, Taatgen, Lebiere, & Krems, 2011). On the other hand, information that is present in the outside world eliminates the need of keeping information in mind (Gray & Fu, 2001). This raises the question whether the process of abductive reasoning changes if less relevant information does not decay because it can be stored in external memory (Gray & Fu, 2001; O’Regan, 1992) and, therefore, remains active without using working memory capacity.

Previous research suggests that people consider present information first (O’Regan, 1992), for instance, information presented on a computer screen. The total effort to handle information is a sum of the effort put into motor action (such as eye movements) and storage and retrieval in memory (Gray & Fu, 2001). As mere eye movements in a limited visual array do not pose high requirements to the motor system, we assume that people prefer getting information using small eye movements from the outside world rather than retrieving it (Gray & Boehm-Davis, 2000; Gray & Fu, 2001). People should, therefore, be able to use and integrate more information when they do not have to retrieve it (Ballard, Hayhoe, Pook, & Rao, 1997; O’Regan, 1992; Spivey & Dale, 2011). Integrating information means that all previous information and more important explanations are taken together to find the least complex explanation for all observations. In contrast, when demands exceed working memory capacity, people tend to explain each observation separately without taking previous information into account. However, this results in a more complex overall explanation (Johnson & Krems, 2001). Further, as the situation model grows with each new piece of information and retrieval requires more resources than inferences from givens, it should also take more time to reconstruct a complex situation model that includes a number of observations from memory rather than from an external memory store.

Taking all these findings together, we can say that the situation model (a) is stored in working memory (b) as a result of limited capacity, (c) is task-dependent, (d) is crucial to the outcome of reasoning, and (e) might influence the processes of reasoning as it determines the amount of information considered.

### Visual attention

The first point above (a), that the situation model is most likely held in working memory, is particularly important when investigating the content and structure of the situation model to make more elaborate assumptions regarding the information used in the reasoning process (e). As we know from a large body of literature, working memory is closely connected with visual attention, which is often reflected in eye movements (Belopolsky & Theeuwes, 2009; Huettig, Olivers, & Hartsuiker, 2010; Theeuwes, Belopolsky, & Olivers, 2009). As attention precedes eye movements (Deubel & Schneider, 1996) and, therefore, determines what we look at next, it influences what is stored in working memory (Theeuwes, Belopolsky, & Olivers, 2009). Also, within the mental representation that is held in working memory, shifts of attention occur (Griffin & Nobre, 2003; Theeuwes, Kramer, & Irwin, 2011) and function as a mechanism to rehearse and maintain information (e.g., Godijn & Theeuwes, 2012).

In essence, attention determines what is part of the mental representation. As one of the key functions of attention is orienting in visual stimuli (Posner, 1994), the stimulus and its complexity should also affect what is part of the mental representation. The amount of present information should not only affect where attention is guided, but also the amount of information that is integrated into the situation model, as the task determines what is processed within and across gaze positions (Hayhoe, Bensinger, & Ballard, 1998). Taken together, manipulations of the task with regard to the amount of given information influence the reasoning process as it guides attention, which in turn determines what information enters the mental representation in working memory. As attentional shifts manifest themselves in eye movements, gaze data are able to shed light on the question of what information is used as we engage in abductive reasoning.

### Research objectives

Following the study of Ballard, Hayhoe, and Pelz (1995), we expect that people experience more difficulties when information has to be retrieved from memory than when information is given or can be derived from givens. As a consequence, study participants should experience the task as more demanding. Thereby, workload was operationalized with eye tracking as behavioral data. We assume that retrieval requires more cognitive resources than inference or gathering information from a visual setup. As eye movements to empty information locations are more enhanced when memory demands are high (Kumcu & Thompson, 2018; Scholz, Mehlhorn, Bocklisch, & Krems, 2011), we expect retrieval to result in more pronounced eye movements to information locations.

Further, we expect that study participants use all information provided when seeking an explanation for a set of observations. However, when information has to be retrieved from memory, we expect participants to focus primarily on the information that is most important to the task as retrieval absorbs more resources. This should also have an impact on the outcome of the reasoning process.

In order to grasp differences in abductive reasoning based on the amount of given compared to retrieved information, we investigate three questions:

1. Do participants experience differences in the difficulty of the task based on the amount of currently given information?
2. Does the process of reasoning change when more information is given*?* To be precise, is the number of items integrated into the situation model smaller when those items have to be retrieved from memory in comparison to when these items are present in the visual array?
3. Does the reasoning outcome change because people use more information for an explanation when they do not have to retrieve it?

### This study

To investigate outcomes as well as the reasoning process depending on given or retrieved information, we used the same visuospatial reasoning task as in our previous study (Klichowicz, Strehlau, Baumann, Krems, & Rosner, 2020). The “black box task” (BBX; Johnson & Krems, 2001; Klichowicz, Strehlau, Baumann, Krems, & Rosner, 2020) is a tool that can be used to study the abductive reasoning process in detail. In this task, a box is presented to study participants, who are asked to infer what objects are inside it by interacting with it. The participant’s precise task is to locate a number of atoms hidden by inferring the path of light rays from their observed entry and exit positions. The entry and exit positions of the light rays represent the observations and are fixed and for all participants similar. The path develops as the light rays interact with the hidden atoms. How this interaction takes place is defined by a small number of rules. Assumptions regarding the locations of the hidden atoms represent the explanations. As explanations must be inferred from rules, we are able to trace the generation of *causal* explanations rather than the retrieval of learned associations or past instances stored in memory (e.g., Klahr & Dunbar, 1988; Thomas, Dougherty, Sprenger, & Harbison, 2008). In other words, the task allows us to investigate how the situation model evolves. Over the course of the experiment, we manipulate how many of the observations and explanations remain visible within the visual field of the participants and how much of the information gathered has to be stored in memory.

**Fig. 1:**
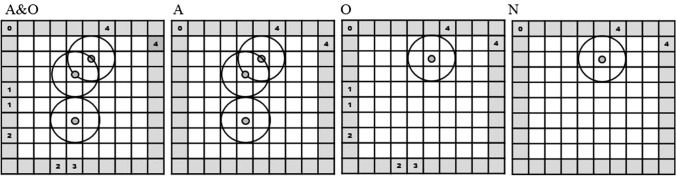
The black box during the last observation of the same trial in the different conditions. In condition A&O, all atoms and observation locations of the former three observations are still visible. Only previously placed atoms remained visible in condition A. In condition O only all previous observation locations were visible. The current observation and the corresponding atom but nothing more remained visible in condition N

In this experiment, we introduced four conditions, which we manipulated in a within subject design. Throughout the trial, all atoms and observations (condition A&O), only atoms (condition A), only observations (condition O), or neither atoms nor observations (condition N) remained visible in the black box display (see Fig. 1). As stated earlier, we were interested how this affects response behavior as well as the process of abductive reasoning itself. The manipulation sheds light on the question of how the situation model changes depending on the amount of information that has to be stored in memory.

### Using eye movements as a method to assess memory retrieval

Eye movements have long been known as a good process measure (Hannula, Althoff, Warren, Riggs, Cohen, & Ryan, 2010; Holmqvist et al., 2011) for processes with information from visually presented givens. Ballard, Hayhoe, Pook, and Rao (1997), for instance, report higher sensitivity of eye movement measures than of conscious reports by study participants.

Further, eye movements are tightly coupled with retrieval processes from memory (e.g., Scholz, Mehlhorn, Bocklisch, & Krems, 2011; Scholz, Mehlhorn, & Krems, 2016). For instance, they play a role in constructing and maintaining the mental image (Brandt & Stark, 1997; Laeng, Bloem, D’Ascenzo, & Tommasi, 2014). Therefore, eye movements are a valuable means of investigating both given and retrieved information. Given information results in spatial indexing, whereas information that has to be retrieved from memory elicits memory indexing. The memory indexing method (Jahn & Braatz, 2014; Renkewitz & Jahn, 2012) utilizes the fact that, when retrieving information, people’s gazes are drawn to the spatial location where the information was previously encoded, even if that information is no longer displayed (see looking-at-nothing phenomenon; Laeng & Teodorescu, 2002; Richardson & Kirkham, 2004; Scholz, Klichowicz, & Krems, 2018; Spivey & Geng, 2001, for an overview see Ferreira, Apel, & Henderson, 2008; Richardson, Altmann, Spivey, & Hoover, 2009). Encoded information is thus stored along with a spatial index (e.g., Kumcu & Thompson, 2016; Pylyshyn, 2001; Richardson & Kirkham, 2004). Probing this information reactivates the associated spatial index, which elicits eye movements toward the (now empty) spatial location.

As visual processing is task-driven (Hayhoe, Bensinger, & Ballard, 1998; O’Regan, 1992; O’Regan & Levy-Schoen, 1983), the amount of given information during a task might result in different eye movement patterns. In the study by Ballard, Hayhoe, and Pelz (1995), display changes affected study participants’ eye movements depending on where they were in the task at that moment, suggesting that vision only considers features that are currently task-relevant.

### Hypothesis 1: differences experienced in task difficulty

People are generally able to engage in abductive reasoning successfully. However, the question remains whether they experience differences in task difficulty even when they are successful. To compare experiences with actual outcomes, we introduce a subjective rating of difficulty of each condition. Following Ballard, Hayhoe, and Pelz (1995), we assume that retrieval poses more demands on participants than acquiring information from the visual setup. This should be evident from the participants’ ratings of the difficulty of conditions. Conditions with visible atom and observation locations (A&O) should be experienced as easiest. We have no assumptions regarding differences depending on whether participants see former explanation (atom) locations (condition A) or can reconstruct them based on observation locations (condition O). However, as retrieval is assumed to be a more demanding process, we expect study participants to rate the condition in which they have to remember observation as well as explanation locations (N) as the most difficult.

### Hypothesis 2: elements of the situation model

As previous explanations are more important for the situation model than previous observations, we predict that study participants spend more time on previous explanation locations than on previous observation locations, regardless of whether explanations or observations are still visible on the screen. Therefore, we assume that participants’ visual attention is driven to previous explanation locations irrespective of condition. If previous explanations are still visible, participants look at them to use the external memory store for the construction of a coherent situation model. If explanation locations are not visible, participants still look at their location as they either construct (if observations are still present) or retrieve (if no previous information is visible) their position in assessing the situation model. As the degree of activation is much smaller for observation locations (Klichowicz, Strehlau, Baumann, Krems, & Rosner, 2020), we assume that participants only look at them when present. Otherwise, the costs of retrieval are too high, as only explanations are relevant for the overall explanation. We expect that participants look at observation locations in order to infer previous explanation locations if previous explanations are not visible.

### Hypotheses 3: integrative solutions

As the external memory store can act as an aid to relieve working memory, Hypothesis 3a proposes that more information is considered to find the best explanation for all observations when information remains visible on the screen. That is, a less complex explanation is used. We call this explanation “integrative” as it integrates previous explanations in order to explain new observations and does not use a new explanation for every observation. Hypothesis 3a, therefore, states that study participants find more explanations that integrate information to a higher degree when more information remains visible and when the setup acts as an external memory store. It is, therefore, irrelevant whether observation locations or explanation locations are still visible. Even though explanation locations are more important for the overall explanation, observation locations can be used to infer explanations rather than to retrieve them.

As a setup that requires keeping all information in memory requires participants to construct, maintain, and retrieve the situation model as needed, we propose that participants take more time to find a coherent explanation in this case. To be more precise, we hypothesize that finding the explanation for the last observation takes more time when atoms and observations have to be retrieved than when information is given (Hypothesis 3b).

## Method

### Participants

For 34 participants, the calibration of the eye tracker succeeded to an accuracy of at least 2° of visual angle. Due to decreasing eye tracking accuracy throughout the experiment, three participants had to be excluded. The remaining 31 participants (17 females, 14 males) were all students from Chemnitz University of Technology and had a mean age of 22.7 years (SD = 3.7). All had normal or corrected to normal vision.

### Apparatus

The task was presented on a 22″ computer screen (1680 × 1050 pixels), which was located at a distance of 63 cm in front of the participants. E-Prime 2.0 was used to present stimuli and participants responded with a standard keyboard and via mouse. At a rate of 120 Hz, a SMI RED remote eye-tracking system sampled data from the right eye during the reasoning task. We used iView X 2.5 to record data following five-point calibration, and BeGaze 3.0 to analyze gaze data with a fixation dispersion threshold of 100 pixels and a duration threshold of 80 ms. Further, we used IBM SPSS statistics 24, Microsoft Excel 2016, JASP 0.8.4.0, and R version 3.4.3 to conduct the analysis.

### The black box task

**Fig. 2:**
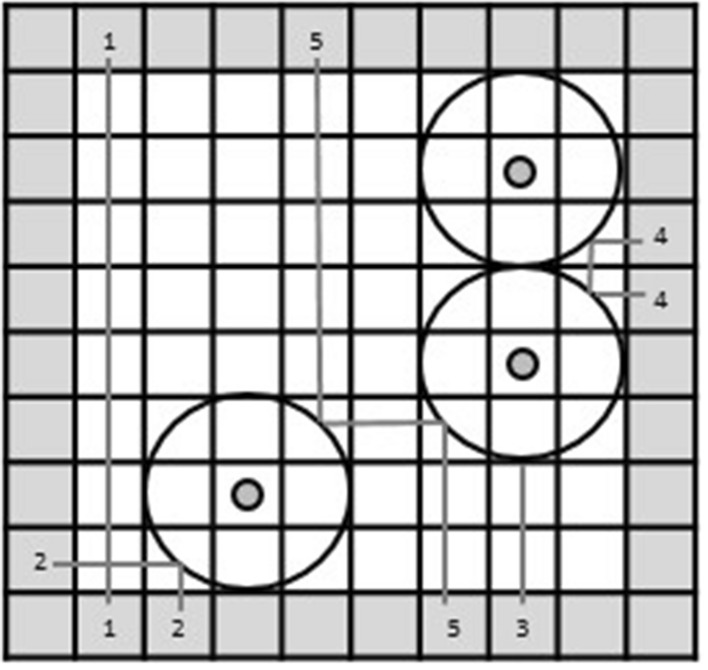
Rules of the black box task (BBX). A light ray entering the black box can take the following paths, depending on where the ray hits the hidden atom: 1 = straight through, 2 = L-pattern, 3 = absorption, 4 = U-pattern, 5 = Z-pattern

The black box task (BBX) was defined by a 10 × 10 grid with a size of 25.92° × 26.27° of visual angle (1015 × 1029 pixels). In this grid, the participants’ task was to locate hidden atoms by following where light rays entered and exited the box. The actual path of the light ray through the box remained hidden from sight. Participants only saw predefined entrance and exit positions of each ray as indicated by a number appearing at the border of the black box (see Fig. 2).^1^

These entry/exit locations are observations according to TAR (Johnson & Krems, 2001) and require one or more explanations, which were operationalized as atoms. Each atom is surrounded by a field of influence (a circle around the atom). As shown in Fig. 2, hitting this field, ray and atom interact according to a set of predefined rules that the participants learned beforehand. Following the numbers that indicated the observation positions of each ray, participants could place atoms using the mouse. Even though participants did not have to place atoms, they were instructed to place each new explanation as early as possible. It was up to participants to decide when to move on to the next trial by pressing the space bar. A digit in the upper left corner of the black box (see Fig. 1) indicated the number of observations left during one trial.

The black box task allowed five different rules, which were as follows: when the ray does not meet any field of influence of an atom, it finds its way straight through the black box. An L-pattern arises if the ray hits the field of influence at an angle and is reflected 90°. A ray of light is absorbed and does not exit the black box if it hits an atom directly in the middle. Combinations of two L-patterns can result in a U- or Z-pattern.

Straight through, L-pattern, absorption, U-pattern, and Z-pattern were the names used to describe the observations for a better understanding of the task. However, the actual observations only consisted of entry and exit locations of the ray. The names of the observed patterns describe the most likely path the ray of light would take through the black box based on the observation locations.

After each presentation of a new observation location, participants were asked to infer and place the atom based on the rules explained above. Participants were instructed to keep the number of atoms to explain a ray pattern as low as possible throughout the trial. Each trial consisted of four observations in sequential order, which were indicated by a number at the entry and exit position of the ray in the BBX (Fig. 2).

All participants solved 12 trials in each of the four conditions with differing amounts of information that had to be stored in memory. In the first condition, all atoms and observation locations remained visible throughout the trial. All information could, therefore, be placed in an external memory store. The conditions were named based on the items that remained visible. Since atoms and observation locations remained in the first condition, it was referred to as A&O. In a second condition, only already placed atoms remained visible (condition A). During the 12 trials of the third condition, only observation locations of the rays remained visible and atom locations had to be remembered (condition O). Because the last condition was completely memory-based and “nothing” remained in the external memory store, it was referred to as N for nothing. All trials consisted of four observations; therefore, Fig. 1 shows what was presented during observation four in each condition. Even though we used the same example for better understanding in Fig. 1, participants did not solve the same trials in each condition, but slight variations that were balanced in complexity and difficulty to prevent learn effects.

**Fig. 3:**
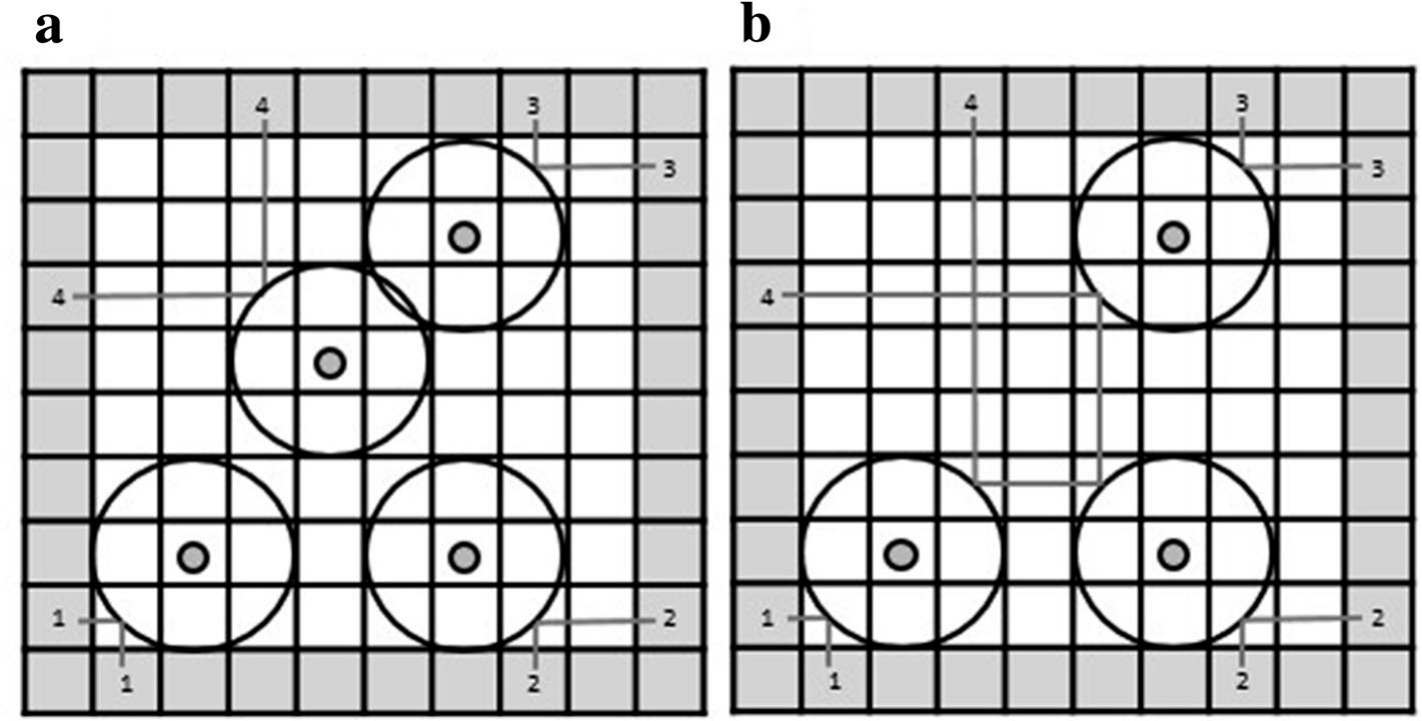
The same trial of the black box solved non-integratively (**a**) and integratively (**b**). To facilitate understanding, we present the difference in the A&O condition with the ray path visible. However, trials of all conditions could be solved both ways, and ray paths were never presented to the participants

Even though participants were instructed to include a preferably small number of atoms in the final explanation of the trial, two-thirds of the trials could be solved in two different ways. In the following, these trials are called experimental trials. First, participants could explain each observation separately without considering previous atoms (Fig. 3a). Second, participants could keep the number of atoms low by using previously set atoms to explain the last observation (Fig. 3b). Because this means that all other atoms had to be integrated to find the explanation, we call the trials that were solved “integrative”. The remaining third of trials during the test phase are called distractor trials and had only one correct solution.

### Procedure

In an initial instruction phase, participants learned the rules of the black box. During this phase, they learned the paths the ray of light took through the black box according to their observation location. Therefore, they first saw a screen explaining the rules much like in Fig. 2, but with one rule at a time. Participants then solved two versions of explained rule (i.e., two absorptions) and received feedback afterwards. If an atom was missing, it appeared blue, if one was in the wrong location, it was red, and a correctly placed atom was green. Participants were only allowed to move to the next rule when each version of a rule was solved correctly. After participants worked through all rules, they saw a screen summarizing them (as in Fig. 2).

During the test phase, participants solved 12 test trials in each of the four conditions (see Fig. 1). The order of conditions was balanced according to a Latin square across participants. That is, each condition was the first, second, third or fourth block for a quarter of all participants. Each block consisted of a practice trial and 12 fixed trials, which E-Prime presented in randomized order. Each block started with a screen explaining the setting in the current condition. That is, what information remained in the visual setup as a trial moved on to the next observation. Afterwards, a five-point calibration of the eye tracker was followed by a practice trial. As in the instruction phase, participants received feedback after the practice trial and had to repeat the trial until it was solved correctly. After the participant run through all 12 test trials of a condition, he moved on to the next one. Between the conditions, there was a standard break lasting three minutes, during which participants were allowed to move freely in the room to prevent a loss in concentration or effects of fatigue. In a last step, following the four blocks representing the four conditions, participants worked through pairwise comparisons rating the task difficulty of the conditions.

### Pairwise comparisons

After participants worked through all four conditions, we asked them to rate the conditions in the form of pairwise comparisons in a paper-and-pencil setup. We contrasted the conditions in pairs, playing through all possible combinations (e.g., condition 1 “atoms and entry/exit position visible”—condition 2 “nothing visible”). We asked participants to highlight the condition that was experienced as more challenging in each of the resulting six pairs. Participants were given an example to make sure they understood the task to highlight the more challenging condition in each pair. Further did we provide an overview presenting each condition on a separate sheet to assure that participants remembered conditions right.

## Results

### Performance

A trial was solved correctly if all atoms were placed according to the rules participants had been given without creating any contradictions. Trials that were solved integratively (that is, participants kept the number of atoms low by using previously set atoms to explain the last observation; see Fig. 3b) as well as those that were solved by placing another atom were counted as solved successfully (see Fig. 3a). This measure is called “accuracy” (ACC). The percentage of experimental trials solved integratively is referred to as ACC-i. Participants solved 85.5% of the trials correctly and 12.8% out of all experimental trials integratively. Even though the Greenhouse–Geisser corrected ANOVA shows significant results indicating a higher accuracy solving the trials when more information was given [*M*_*A&O*_ = 92% (SD = 13); *M*_*A*_ = 90% (SD = 11); *M*_*O*_ = 86% (SD = 15); *M*_*N*_ = 79% (SD = 22); *F*_*ACC*_ (2.02, 60.46) = 4.52, *p* = 0.02, *η*_p_^2^ = 0.13, BF_10_ = 10.99], there are no significant differences in the Bonferroni pairwise comparisons between conditions regarding the percentage of trials solved correctly. As this shows that participants were generally able to solve the task across conditions, in the following we will focus on *how* the task was solved. We will take a closer look at trials that were solved integratively in Hypothesis 3a.

Participants needed on average *M* = 11.48 s (SD = 3.27) to work on an observation. As participants themselves decided when to move on to the next observation, the time participants worked on an observation is the time the observation could be viewed on the screen. This measure is, therefore, called “viewing time” (VT).

It took on average *M* = 45.44 s (SD = 13.05) to solve a whole trial. The time participants needed to solve a trial will be called “time” (T).

There were no differences between conditions, either in the processing of an observation [*F*_*VT*_ (3, 90) = 1.66, *p* = 0.18, *η*_p_^2^ = 0.05, BF_10_ = 0.29] or in the time participants took for an entire trial [*F*_*T*_ (3, 90) = 1.44, *p* = 0.24, *η*_p_^2^ = 0.05, BF_10_ = 0.22].

As participants solved all four conditions in four blocks, presented in randomized order, it is important to look at carry-over effects due to learning (see Table 2). Data show that across all of the conditions in the time participants needed, only Block 4 is significantly faster than Blocks 1, 2, and 3 [*F*_*VT*_ (3,90) = 11.44, *p* < . 001, *η*_p_^2^ = 0.28, BF_10_ > 1000; *F*_*T*_ (3,90) = 10.50, *p* < 0.001, *η*_p_^2^ = 0.26, BF_10_ > 1000]. Both viewing time (VT) and time (T) show significant results for the Bonferroni pairwise comparisons of Block 4 with each of the other three blocks. Accuracy even seems to drop slightly over time. However, the decrease in ACC is not a significant statistical result [*F*_*ACC*_ (1.70, 51.03) = 2.01, *p* = 0.15, *η*_p_^2^ = 0.06, BF_10_ = 0.53]. None of the Bonferroni comparisons yields a *p *value below 0.05. When looking at the conditions independently, VT and ACC also show no significant change over time (all *p*s > 0.05, all BF_10_ < 3). That is, none of the conditions show significantly differing results depending on the point in time they were presented throughout the experiment. As for the time participants needed to solve all four observations, the ANOVA yields a significant result for the condition in which atoms as well as observation locations remained visible [*F*_*T.A&O*_ (3, 34) = 3.86, *p* = 0.02, *η*_p_^2^ = 0.25, BF_10_ = 4.44], indicating that participants solved trials faster when this condition was presented later in the experiment. Regarding all other conditions, ANOVAs show no meaningful results (all *p*s > 0.05, all BF_10_ < 3).

**Table 2:** Possible learning effects over the course of the experiment measured in the averaged time participants worked at an observation, participants needed to solve a trial as well as accuracy and the amount of trials solved integratively

	Viewing time in ms		Time in ms		Accuracy in %		Trials solved integratively in %	
	*M*	SD	*M*	SD	*M*	SD	*M*	SD
Block 1	12,060	3077	48,042	12,138	92.6	9.8	8.5	20.5
Block 2	13,014	3292	50,741	13,218	85.4	13.7	13.3	26.2
Block 3	12,804	3582	51,188	14,342	84.7	14.1	15.3	32.5
Block 4	10,143	2458	40,258	9850	83.7	24.0	16.7	34.5

Even though participants solved trials increasingly integratively, none of the comparisons reach significance. In conclusion, even if participants became slightly faster over time, there are no meaningful differences based on the order in which conditions were presented. As a result, for all further analysis, we collapsed data over blocks.

### Gaze analysis

**Fig. 4:**
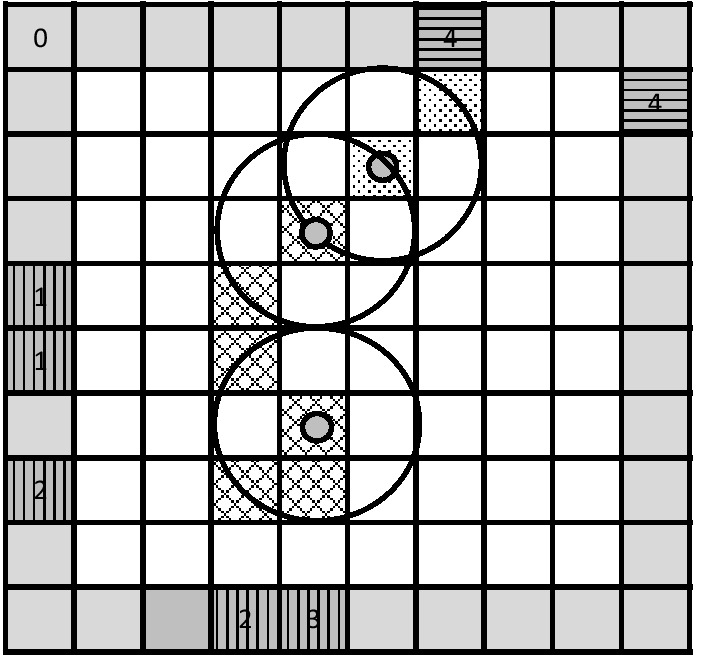
AOI definitions used to analyze the data.  = current observation locations;  = current atom and location at which the ray hit the field of influence of the current atom;  = previous atom locations and  = previous observation locations. This figure shows an example of the A&O condition. AOIs were marked analogously for the remaining three conditions

To analyze eye movements, each grid square was defined as one AOI. This resulted in 100 separate AOIs that were each sized 2.64° × 2.64° of visual angle (102 × 102 pixels). We coded only relevant AOIs for further analysis (Fig. 4). AOIs defined as relevant were those marking the entry/exit locations of the rays, the field where the rays hit an atom’s field of influence, as well as the AOIs where atoms should be placed according to the rules of the BBX. We combined the field where the ray hit the field of influence and the AOI with the actual atom location into a category labelled “atom”.

We analyzed gaze data for each new observation presentation separately. The current observation was labelled as “current observation location” and the current atom was labelled “current atom” as compared to “previous observation locations” and “previous atoms”, which were the information locations of all already seen observations throughout a trial. To differentiate among former atom and observation locations, we also used the point in time in the trial when the information was presented. Therefore, the first observation (observation location) is coded O1 and the corresponding atom A1. Following this, O2 and A2 represented the second, and O3 and A3 the third observation.

Gaze data are defined by fixation times in milliseconds to the different AOIs. Because participants determine the amount of time they spend on each observation freely, fixation times were divided by the time spent by the participant to watch an observation (VT). As a result, we worked with proportions of fixation times (FT). Irrelevant AOIs served as a baseline measure. For each trial, irrelevant AOIs from the grey border area were defined to compare with the observation locations, and irrelevant AOIs from the white grid area were defined to compare with the atom locations. We thus selected a random AOI that never contained any observation location or atom or field of influence throughout the trial.

Gaze analysis contains only data from trials solved correctly, as we are interested in the memory indexing or spatial indexing when reasoning is successful.

### Hypothesis 1: differences experienced in task difficulty

Descriptive data show that only a small number of people rated condition A&O as more challenging as the other conditions (Table 3, see column A&O). The majority of participants rated the condition in which atoms as well as entry/exit positions had to be remembered (N) as more challenging as all other conditions (Table 3, see column N).

**Table 3:** Ratings of difficulty between conditions. The numbers represent the amount of participants that rated the condition in the top row as more challenging than the condition presented in the first column

	A&O	A	O	N
A&O		27	28	29
A	4		13	28
O	3	18		27
N	2	3	4	

Using the BradleyTerry2 package in R (Turner & Firth, 2012), we calculated a Bradley–Terry model fit. This model is a logistic model for paired choice data and provides evidence on participants’ choice between a number of attributes or objects by pairwise comparison of all attributes (for a detailed explanation of the model see Agresi, 2007; Bradley, 1984). It, therefore ,shows how people perceive the difficulty of each condition, irrespective of their success in solving it. We set the condition “atoms and observations visible” (A&O) as a baseline and found that participants rated the condition “atoms visible” (A) as more difficult, with a parameter of 1.94, the condition “observations visible” (O) with a parameter of 1.81, and condition “nothing visible” (N) the most difficult with a parameter of 3.74.

Translated into probabilities (see Agresi, 2007 p. 266), participants rated with a probability of 0.98 the condition A&O easier as condition N, with a probability of 0.86 easier as O, and with a probability of 0.87 easier as condition A. Condition O is rated easier as condition A with a probability of 0.47. Both conditions A and O are rated easier as condition N with high probabilities of 0.86 and 0.87. This confirmed our hypothesis that participants experienced conditions A and O as similarly challenging, and condition N, where everything had to be retrieved, as much more difficult. This lends support to our hypothesis that retrieval poses subjectively greater demands than reconstruction.

### Hypothesis 2: elements of the situation model

**Fig. 5:**
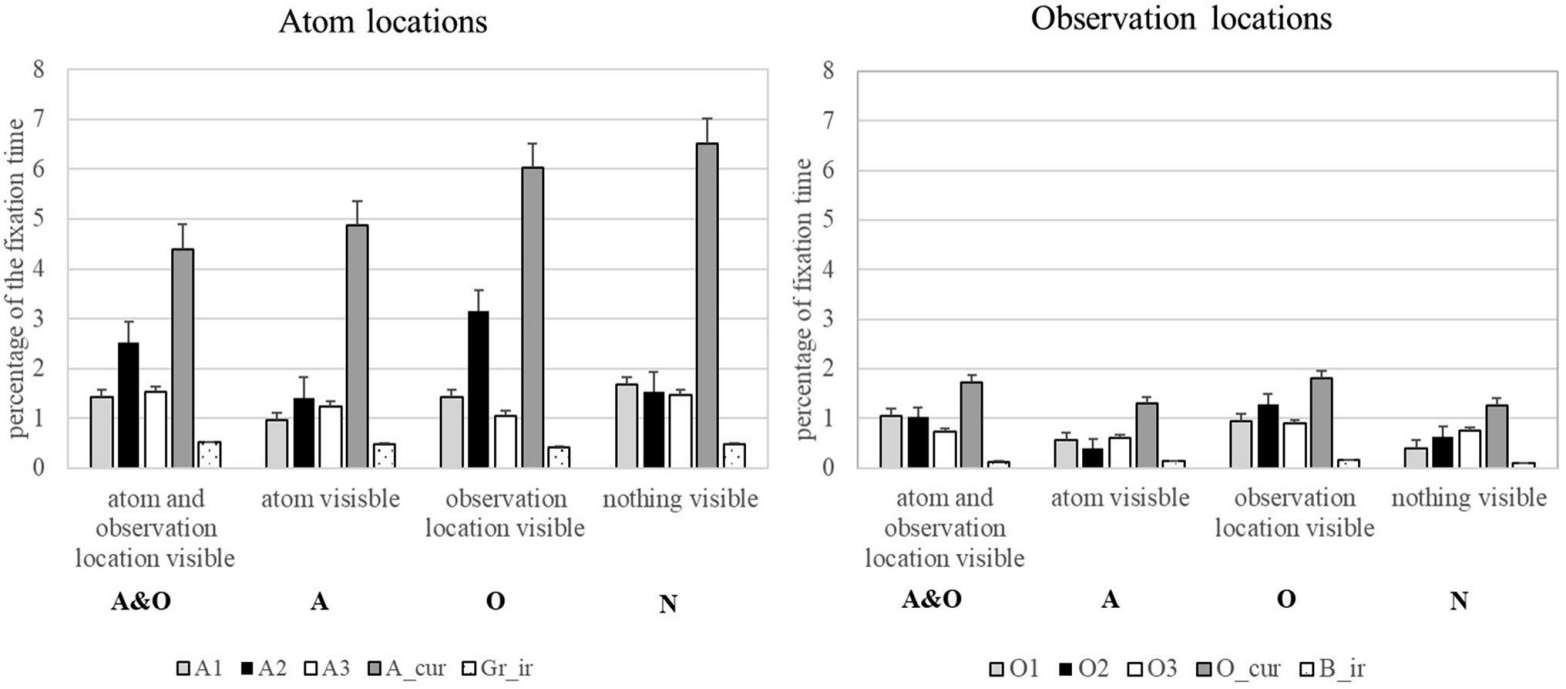
Overview of gaze data. Fixations to atom locations (left) and observation locations (right) depending on their point of presentation in the trial. A1/O1 stand for the first observation and corresponding atom; analogously, A2/O2 and A3/O3 stand for the second and third observation and corresponding atoms. A_cur/O_cur mark the currently presented information. Gr_ir stands for randomly chosen irrelevant fields in the white grid of the BBX and B_ir for randomly chosen irrelevant fields in the grey border. Error bars represent standard errors

In the overview summary graphic presented in Fig. 5, participants spent more time looking at each previous atom location than at any irrelevant field of the grid. At the same time, they paid more attention to each previous observation location than to any randomly chosen irrelevant field in the grey border area. However, current observations as well as their corresponding explanations (atoms) were always looked at most by the participants. Generally, explanations seem to play a much greater role than the location of observations. It is interesting to note that in this initial overview, all conditions seem to form the same pattern: no change in strategy depending on reasoning from givens vs. reasoning from memory.

To illustrate that participants look to all atom and observation locations, irrespective of whether they are currently visible on the screen, we calculated a Greenhouse–Geisser corrected 2 (object of gaze: atom, observation) × 3 (information type: current, previous, irrelevant) repeated measures ANOVA for the condition N. We choose this condition where participants had to retrieve all previous information from memory because it demonstrates that participants engaged into memory indexing. The analysis revealed a main effect for object of gaze, [*F* (1, 30) = 85.38, *p* < 0.001, *η*_p_^2^ = 0.74] and information type [*F* (1.19, 35.63) = 78.80, *p* < 0.001, *η*_p_^2^ = 0.72]. The first main effect indicates that explanation location receive significantly more attention (higher proportion of fixation times) than observation location. The second main effect shows that participants looked more to current information location (because they are visible on the screen) than to previous information location and irrelevant spatial areas. That is, data support a step of comprehension and integration into a situation model of new information as assumed by TAR (Johnson & Krems, 2001; see also Klichowicz, Strehlau, Baumann, Krems, & Rosner, 2020). Even more important is that participants looked also more to previous information location (that no longer contained visible information) than to irrelevant spatial areas. This indicates that the information is still part of the mental representation. The differences between looks to current, previous, and irrelevant areas are also supported by post–hoc Bonferroni pairwise comparisons (all *p*s < 0.001).

To test our hypotheses regarding the gaze data, we calculated repeated measures ANOVA with the factors atom (visible/not visible), observation location (visible/not visible), and object of gaze (previous atom locations/previous observation locations).

The ANOVA yielded three main results and no interactions. The main effect for object of gaze [*F* (1, 30) = 53.33, *p* < . 001, *η*_p_^2^ = 0.64, *BF*_*10*_ > 1000] indicates that participants spent more time looking at the atom locations than at the observation locations regardless of what was visible on the screen. This favors the hypothesis that explanation locations are more important than previous observation locations in the process of abductive reasoning. This is further supported by the fact that there is no significant difference between the time participants spent looking at atom locations depending on whether atom locations are still visible or have to be retrieved. As the absence of significance does not provide statistical support, note that the BF_01_ speaks in favor of the null hypothesis [Raftery 1995; *F*(1, 30) = 0.16, *p* = 0.70, *η*_p_^2^ = 0.005, BF_01_ = 6.82]. It follows that data show no statistical difference between memory indexing and spatial indexing referring to the atom location. Especially the value of the Bayes Factor BF_01_ supports this suggestion.

The third main result concerns the observation location. A significant result [*F* (1, 30) = 12.76, *p* = 0.001, *η*_p_^2^ = 0.30, *BF*_*10*_ = 44.15] supports the hypothesis that participants only look at observation locations when they are visible.

To summarize, these results speak in favor of the assumption that the explanation locations are most important and that they are part of the situation model irrespective of memory costs. Observation locations, on the other hand, are only included when they can be stored in external memory.

### Hypothesis 3: integrative solutions

Hypothesis 3a stated that participants find more integrative explanations when more information remains visible. That is, people use more previous information to find an explanation when the setup acts as an external memory store. However, there were no significant differences in the number of trials solved integrative between the four conditions [*F* (2.36, 70.67) = 0.57, *p* = 0.59, *η*_p_^2^ = 0.02, BF_01_ = 12.07]. When all explanation and observation information stayed visible, 14% (SD = 30) of the test trials were solved integrativley. With 16% (SD = 30) when only atoms remained and 13% (SD = 30) when only observation locations stayed visible, all conditions produced more integrative solutions than the strictly memory based one (*M*_*N*_ = 11%; SD = 25). Even though this result is in the expected direction, none of the Bonferroni pairwise comparisons between conditions are statistically meaningful.

As a setup in which all information must be kept in memory requires participants to construct, maintain, and *retrieve* the situation model as needed, we proposed in Hypothesis 3b that participants take more time to find coherent explanations for the last observation. A repeated measures ANOVA revealed no differences between conditions for the amount of time participants need to respond to the last observation [*F* (3,90) = 1.21, *p* = 0.31, *η*_p_^2^ = 0.04, BF_01_ = 4.13]. Participants needed more time for the last observation *M*_*N*_ = 6.3 s (SD = 3.5) when nothing remained visible compared to when atoms and observations (*M*_*A&O*_ = 5.0 s, SD = 1.9), atoms (*M*_*A*_ = 5.5, SD = 2.3) or observation locations (*M*_*O*_ = 5.2, SD = 2.2) remained visible. Even though this shows a trend in the right direction, none of the pairwise comparisons yields significance (all *p*s > 0.05).

Concluding, given this sample size and task, people show no reliable differences concerning information integration or time to solve a trial depending on the amount of given information.

## Discussion

According to TAR (Johnson & Krems, 2001), when seeking the best explanation for a number of observations, people have to construct an understanding of the current situation, which is represented as a situation model. The complexity and nature of this representation depends on cognitive resources as much as on the current task. Because the situation model is crucial to successful reasoning, this study was interested in how the process as well as the outcome of reasoning changes based on the amount of given information. In a sequential abductive reasoning task, we manipulated whether previous observations as well as previously found explanations remained visible throughout a trial. Employing eye tracking, we were able to assess the information used to find the best possible explanation for a set of observations. This allowed us to include memory retrieval of information (based on memory indexing; Renkewitz & Jahn, 2012) as well as the assessment of information from the external world. We were not only interested in changes regarding the process but also the experienced difficulty between conditions. Therefore, we also employed pairwise comparisons in our study.

In a last research question, we were interested in whether manipulations of the information available had an impact on the reasoning outcome. More precisely, we investigated whether given information leads to more complex information integration to reach a solution compared to information that has to be retrieved from memory. Manipulation of the amount of information held in memory during abductive reasoning had not been done before, in particular, not in close relation to eye tracking as a process-tracing measure.

### Differences experienced in task difficulty

Our results show that when finding an explanation for a set of observations, people experience less difficulty when information can be gathered from the external world rather than being retrieved from a mental representation. It thus makes a difference whether needed information (e.g., previously found explanations) is assessed directly from the visual array. However, in our study, the mere evaluation of task difficulty does not have an impact on the actual outcome of the reasoning process, as people do not show more integrative solutions for conditions rated as easy and do not produce more or faster solutions. We assume that participants simply optimize the reasoning process by prioritizing more important information. That is, information that is not crucial to the reasoning outcome, such as already explained observations, is neglected.

### Elements of the situation model

Our results reveal that participants pay attention to previous atom locations irrespective of whether they are still visible in the visual array or have to be retrieved from memory. This is in line with our hypotheses. As previous atom locations represent previously found concrete explanations, they are crucial to the overall explanation and have to be represented in the situation model even if they have to be stored in memory. This is also in line with research on decision making, which found no differences in strategy applications between decisions from memory and decisions from givens (Rieskamp & Otto, 2006). However, according to our data, this holds true only for information that is directly relevant to the task. As observation locations decline in importance once they are explained, they are only part of the situation model when still present. It may be that people simply look at objects that are presented in the array. However, this would not explain why participants look at absent explanation locations. We do not believe that mere salience or bottom-up processes in perception lead to gazing toward previous observation positions, as we assume that gaze patterns are not only salience-driven, but also goal-driven (e.g., Ballard & Hayhoe, 2009). It is more reasonable to assume that participants know what kind of information has to be included in the situation model and what information matters but can be left aside if the costs are too high. Therefore, eye movements reflect not only memory processes but also adaption to a task that is not necessarily visible in reasoning outcomes. This is an indicator that the results of Ballard, Hayhoe, and Pelz (1995) that people only engage in active memorization and retrieval when necessary can be applied to reasoning. Please note however, that this study only shows that eye movements and retrieval are closely intertwined (Hollingworth, 2005, 2006; Renkewitz & Jahn, 2012; Spivey & Geng, 2001). It does not allow any conclusion whether eye movements can act as an aid to retrieval (Anderson, Bothell, & Douglass, et al., 2004; Scholz, Mehlhorn, Bocklisch, & Krems,^2011^; Scholz, Klichowicz, & Krems, 2018; Scholz, Mehlhorn, & Krems, 2016).

In summary, our results show that explanation locations are much more relevant than observation locations when seeking the overall explanation. Eye movements are not just automatically driven to salient information but represent the content of the situation model. Data show that participants are able to construct a mental representation using information both from memory and from the outside world, which is also in line with previous research (Hayhoe, Bensinger, & Ballard,^1998^). In this context, our data suggest that the situation model can be constructed from both, information stored in memory and information from an external memory store. This is especially the case when participants perceive a task as demanding.

### Integrative solutions

As we expect people to change strategies when a task is more demanding with respect to working memory, we cannot say whether the actual reasoning outcome changes as well. It is possible that even if keeping information in memory is very costly, participants remember information that is crucial to the process independent of the fact that it is still present because it is a safe strategy to good performance (Gray & Fu, 2001). It is also possible that the change in gaze strategy is able to compensate for the higher retrieval demands. However, in our study, participants generally solved only a small number of experimental trials integratively. Future research should focus more on the circumstances that lead to global integrative solutions. In our study, the observation that could be solved integratively was always an L-pattern. Even in the study by Johnson & Krems, (2001) this pattern did not often result in integrative solutions. Different patterns such as absorptions, which can only be explained using previous explanations, might elicit a different response pattern. Here, we avoided absorption for integrative solutions in order to investigate people’s behavior when they choose how to solve a trial. Our results therefore do not contradict TAR (Johnson & Krems, 2001), as according to the model, explanations that are not yet concrete lead to integrative solutions.

TAR (Johnson & Krems, 2001) predicts that otherwise, people use the simplest explanation possible. Because Johnson and Krems (2001) could not support this prediction with their data, our study helps to shed light on this question. In summary, people use easy (non-integrative) solutions when resources are not sufficient to integrate all explanations. That is, when demands on memory are too high, people do not retrieve and combine the information needed to find a solution of a new observation based on already existing explanations but create an entirely new explanation. This leads to the conclusion that our task was challenging even when all information remained visible. Increasing difficulty based on more information that had to be stored in memory led to a further decrease in the percentage of integrative solutions. However, this result was not of statistical significance, which can be explained by compensatory strategies such as the use of functional eye movements and the neglect of less important information. This is also evident in the fact that the time participants needed to solve the last observation and therefore to retrieve a complex situation model involving three observations and explanations did not change significantly based on the amount of information given.

In order to provoke differences in reasoning performance, future research should introduce a secondary task. To solve the BBX task, participants need to integrate already seen observations and explanations. As this process most likely takes place in the spatial component of working memory (visuospatial sketchpad; see Baddeley & Hitch, 1974, 1994), we propose a spatial task such as finger tapping of complex patterns as a secondary task. There is already evidence that this procedure has an influence on combining visual material (e.g., Pearson et al. 1999). Therefore, this approach, coupled with eye tracking might not only provoke different reasoning outcomes, but might even be able to identify phases during the process of abductive reasoning where working memory demands are especially high.

## Summary

This study provides evidence of two things: first, reasoning is based on a mental representation that can be constructed from memory and outside sources alike. People thus experience construction from given instances as much less demanding, even if their success in the reasoning task does not show differences. Second, differences in task difficulty are also evident in changes in the situation model when more information has to be retrieved from memory. If more information has to be retrieved, changes concerning the process (i.e., the content of the situation model) occur first. Participants restrain themselves only to include most important information into the mental representation when retrieval demands are high. In our study, this most important information consists always of already found explanations. Observations that are already understood in a sense that the reasoner can explain them are only included when memory demands allow it. Therefore, the task influences how abductive reasoning takes place but not necessarily, whether it is successful or not.

## Footnotes

1 All experimental materials and data are available at https://osf.io/b2yhx/.
